# Continuous Variables Graph States Shaped as Complex Networks: Optimization and Manipulation

**DOI:** 10.3390/e22010026

**Published:** 2019-12-24

**Authors:** Francesca Sansavini, Valentina Parigi

**Affiliations:** Laboratoire Kastler Brossel, Sorbonne Université, CNRS, ENS-PSL Research University, Collège de France, 4 Place Jussieu, F-75252 Paris, France; francesca.sansavini@lkb.upmc.fr

**Keywords:** continuous variables clusters, complex quantum networks, quantum routing

## Abstract

Complex networks structures have been extensively used for describing complex natural and technological systems, like the Internet or social networks. More recently, complex network theory has been applied to quantum systems, where complex network topologies may emerge in multiparty quantum states and quantum algorithms have been studied in complex graph structures. In this work, we study multimode Continuous Variables entangled states, named cluster states, where the entanglement structure is arranged in typical real-world complex networks shapes. Cluster states are a resource for measurement-based quantum information protocols, where the quality of a cluster is assessed in terms of the minimal amount of noise it introduces in the computation. We study optimal graph states that can be obtained with experimentally realistic quantum resources, when optimized via analytical procedure. We show that denser and regular graphs allow for better optimization. In the spirit of quantum routing, we also show the reshaping of entanglement connections in small networks via linear optics operations based on numerical optimization.

## 1. Introduction

In the last few decades, network theory has provided a natural framework for describing complex natural, social, and technological structures [1,2]. Recurrent types of complex networks, like the scale-free networks, have been recovered in phenomena at different scales, where the functionality of the systems seems to be closely related to their structure [3,4]. More recently, complex networks have gained attention in the quantum realm, where several theoretical works [5,6,7,8,9,10] show that complex structures may play a role in quantum information technologies. It is clear that, as quantum architectures are reaching larger scales, their internal arrangement starts to play a significant role in their functionality. Moreover, network structures are clearly at the base of quantum communication protocols [11], but also appear in particular kinds of multiparty entangled states that allow for measurement-based quantum computing (MBQC) protocols [12].

Recently, new records have been established in superconductor [13,14,15] and Rydberg [16,17] based technologies, and extremely large entangled states have been generated in the optical domain [18,19,20,21]. We can describe superconducting and Rydberg platforms as networks of interacting qubits, where quantum information is encoded in Discrete Variables (DV) states. Differently, in the optical domain, networks of entangled optical modes are deterministically generated and measurement-based quantum protocols can be implemented via a Continuous Variables (CV) encoding [22]. The control on the reconfigurability of the cited networks is gradually increasing and, in particular, in the CV case, it is possible to have totally reconfigurable networks (with all-to-all connections) mimicking complex networks structures [23].

Here, we study CV entangled states, named CV graph states or CV cluster states, arranged with shapes that are typical of real-world complex networks, in order to investigate their properties for quantum computing or quantum networking (communication) protocols. CV graphs have been introduced in the context of measurement-based quantum computing exploiting CV resources [22,24]. While usually the name “cluster” is used when the graph shape allows for universal quantum computing, in this work, we will use the terms “cluster” state and “graph state” as synonyms. In CV-quantum computing, quantum information is encoded in variables that can take a continuum spectrum of values. In optics, these variables are the quadratures of the electromagnetic field (called amplitude and phase quadrature) and they correspond to the position *q* and momentum *p* of the harmonic oscillator, which represents the behavior of a single mode of the field. Ideal cluster states, which involve perfect correlations between quadratures of different optical modes, cannot be reached experimentally, as they would require an infinite amount of energy (squeezing) to be established. Thus, experimental clusters always involve a poorer degree of correlations, which cause errors and noise in computation. Given experimentally accessible resources, analytical optimization protocols have been proposed to choose state manipulations that better distribute the correlations in order to minimize errors [25], then improve the quality of the cluster. In this work, we apply the optimization procedure to complex cluster shapes and we find that denser and more regular network shapes allow for better quality [26].

As the classical internet can be described via a complex network model, it is worth considering complex network shapes in quantum communication protocols. In particular, we are interested in quantum routing, i.e., the establishment of a direct entanglement link between two arbitrary nodes of a network in order to have an exploitable channel for quantum teleportation. Quantum routing protocols have been mainly studied in the DV approach [27,28], while here we study the particular setting of CV quantum resources. In particular, instead of considering a set of initially disconnected nodes subjected to routing via local operations, we consider a network of CV entanglement correlations, which can be easily produced with the current technology [18,19,20], and then we reconfigure it via partially-local and easy operations (linear optics). The optimal operations are obtained via numerical optimization. We found solutions for small networks of different geometry which are shared between two parties that are allowed to act via local linear optics operations in their set of nodes [26].

## 2. Results

### 2.1. Background: Cluster States and Complex Networks

Ideal cluster states are built starting from a set of modes of light, which are placed in the zero-momentum eigenstate ${|0\rangle }_{p}$, i.e., infinitely squeezed vacuum states along the momentum quadrature *p*. Entangling $C_{Z}=\exp\left(ı{\hat{q}}_{i}⊗{\hat{q}}_{j}\right)$ gates are then applied between couples of modes *i* and *j*, according to a given configuration, which can be represented by a graph. A graph is defined by a set {V,E}, where V is the set of nodes (vertices) and E are the edges. In the graphical representation of the cluster, the nodes represent different modes of light, while the edges between the nodes are the entanglement correlations given by the application of the $C_{Z}$ gates. The graph is characterized by the adjacency matrix *V* whose elements $V_{ij}$ are set to 1 when an entangling gate has been applied between the two nodes *i* and *j*, and 0, otherwise.

An ideal cluster state of *N* modes with adjacency matrix *V* is given by

(1) $$|\Psi_{C}\rangle =\prod_{1\leq i<j<N}\exp\left(ıV_{ij}q_{i}⊗q_{j}\right){|0\rangle }_{p}^{{⨂}^{N}}.$$

Cluster states are characterized by a particular set of operators, called *nullifiers*, that read

(2) $${\hat{\delta }}_{i}={\hat{p}}_{i}-\sum_{j\in N(i)}{\hat{q}}_{j},\;\mathrm{such}\;\mathrm{that}\;{\hat{\delta }}_{i}|\Psi_{C}\rangle =0,$$

where ${\hat{\delta }}_{i}$ and N(i) denote respectively the nullifier and the set of the nearest neighbours of the *i*-th node. Cluster states are eigenstates of the nullifiers with zero eigenvalue, so, for an ideal cluster, the following condition holds: $\Delta^{2}\delta_{i}=0$. In addition, being Gaussian states, cluster states are fully described by mean values of quadratures and covariance matrices [29]. By defining the vector $\hat{X}=\left\{{\hat{q}}_{1},\ldots ,{\hat{q}}_{N},{\hat{p}}_{1},\ldots ,{\hat{p}}_{N}\right\}$, cluster states are identified by mean values $\langle {\hat{X}}_{i}\rangle =0$ and covariance matrix σ with elements $\sigma_{ij}=\left(1/2\right)\langle \left({\hat{X}}_{i}{\hat{X}}_{j}+{\hat{X}}_{j}{\hat{X}}_{i}\right)\rangle$.

As already said, in a realistic situation, CV cluster states are always imperfect, due to the impossibility of reaching an infinite amount of squeezing to obtain the zero momentum eigenstate. Approximated clusters, when used in measurement-based quantum computing protocols, at each step of the computation, introduce a certain amount of noise that can be quantified with the variance of the nullifiers of the cluster [22]. Therefore, the smaller the value of $\Delta^{2}\delta_{i}$ is for every node, the better the quality of cluster is.

Instead of acting with $C_{Z}$ gates on multimode squeezed vacuum states, as in the original formulation, cluster states can be implemented via linear optics transformations on the multimode squeezed vacuum state [30,31,32]. This is the implementation that is pursued in experimental setups [18,19,20,21], as it is easier and less costly than the implementation of $C_{Z}$, which requires online squeezing.

Linear optics manipulations are described by unitary transformations *U* on the creation and annihilation operators of the different light modes, so that the annihilation operator of the mode *i* is transformed as ${\hat{a}}_{i}\to \sum_{j}U_{ji}{\hat{a}}_{i}$. This corresponds to orthogonal symplectic transformations *S* acting on the quadratures, which transform the covariance matrix as $\sigma \to S\sigma S^{T}$.

To implement a cluster with adjacency matrix *V*, we can apply the following unitary operation:

(3) $$U=\left(I+ıV\right){\left(V^{2}+I\right)}^{-\frac{1}{2}}O,$$

where I is the identity matrix and *O* is an arbitrary real orthogonal matrix. This orthogonal matrix provides supplementary N(N−1/2) degrees of freedom that can be exploited to optimize given properties of the cluster [25]. In particular, we can optimize given properties of the nullifiers via an analytical protocol [33], with the aim, for example, of reducing their variances, hence improving the quality of the cluster.

In this work, we investigate, for the first time, clusters whose graphical representation {V,E} corresponds to three different models of complex networks [1]: the Barabási–Albert [34], the Erdős–Rényi [35], and the Watts–Strogatz [36]. These models have been developed in graph theory in order to reproduce the behaviors of complex systems. The Erdős–Rényi has been the first model considered for complex networks. It is based on random graph: given a fixed number of nodes *n*, two nodes have the probability $p_{ER}$ to be connected by an edge. The model generates graphs with approximately $p_{ER}n\left(n-1\right)/2$ edges which are distributed randomly. Most nodes have a comparable node degree *k*, defined as the number of edges of the given node.

Later, by looking at the degree distribution of many real-world networks, it has been clear that complex networks go beyond the random graph model, as many networks (like Internet or the WWW) are characterized by a power-law distribution of the degree. Thus, new models, such as the Barabási–Albert, have been developed in order to reproduce the structure of these networks, also known as scale-free networks. Barabási–Albert networks grow from a small number of nodes according to preferential attachment, i.e., the fact that new nodes attach preferentially to nodes that already have a high degree (high number of links). In the growth process, the parameter $m_{BA}$ specifies the number of links coming with the new node. Hubs, i.e., heavily linked nodes, arise spontaneously in this model.

**Figure 1:**
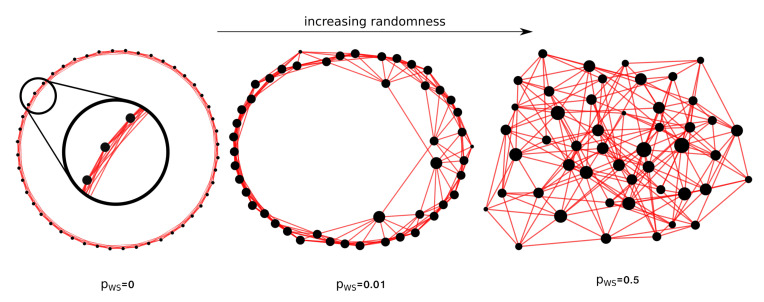
Rewiring of a regular 48-node network for the construction of a “small world” network as shown in [35].

Watts–Strogatz networks lie between regular and random graphs and exhibit small-world properties typical of social networks. They are built from a regular network by rewiring its edges, to increase randomness. From a ring lattice with *n* nodes and *k* edges per node, which connect it to the closest neighbors, each edge is randomly rewired with probability $p_{WS}$. The rewiring parameter $p_{WS}$ spans from 0, the regular graph, to 1, which corresponds to a totally random graph, as shown in Figure 1. Even if we are going to study graphs with a small number of nodes compared to the ones for which these models have been developed, we can assess a different behavior for the corresponding graph states in the three classes.

In the following, we will see how, starting from a given set of modes with finite squeezing values, we can optimize the quality of the clusters corresponding to these complex networks models and how the result of the optimization depends on the parameters of the analyzed model.

### 2.2. Improving the Overall Quality of a Complex Cluster

**Figure 2:**
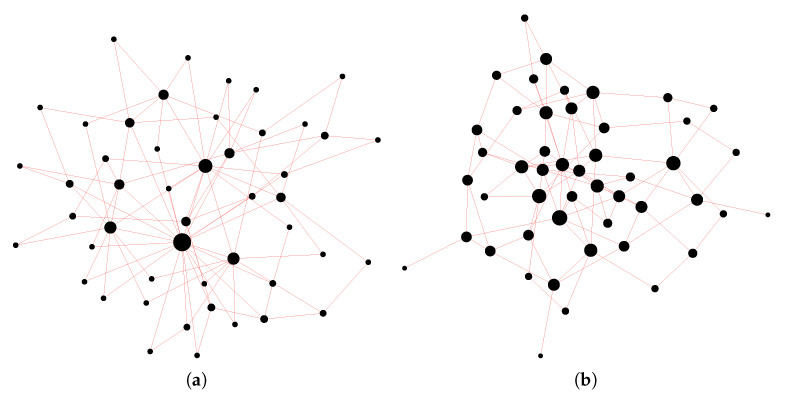
Comparison between two models of complex networks, both with an average degree of 〈k〉∼3.9. The size of the dots increases with the number of links. (**a**) Barabási–Albert model with $m_{BA}=2$, with a maximum node degree of *k* = 22; (**b**) Erdős–Rényi model with $p_{ER}=4/49$, with a maximum node degree of *k* = 8.

We test here cluster states with the structure of the three models defined above: Barabási–Albert (BA), Erdős–Rényi (ER), and Watts–Strogatz (WS), with different characterizing parameters $m_{BA}$, $p_{ER}$, and $p_{WS}$. In Figure 2, the difference between a scale-free network and a random network with a comparable average degree is shown. As we see in Figure 2a, the Barabási–Albert network exhibits highly connected nodes (*hubs*) that are absent in the Erdős–Rényi model (Figure 2b).

**Figure 3:**
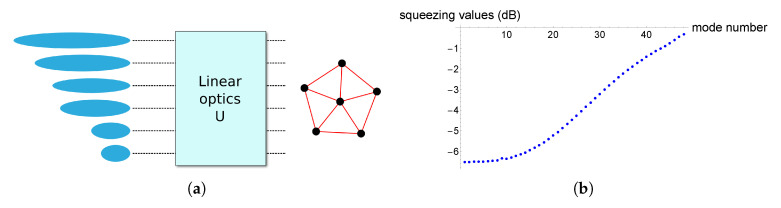
Realistic implementation of complex cluster states. (**a**) implementation of a cluster via a linear optics transformation acting on a series of squeezed modes; (**b**) list of realistic squeezing values of the input modes for the implementation of a 48-mode cluster.

We consider at first the implementation of 48-mode complex clusters. As explained above, the clusters are implemented from a 48-mode squeezed vacuum via the application of the linear optics unitary of Equation (3) corresponding to the graphical shape we want to reach [25]. The scheme is presented in Figure 3a. In order to pursue realistic implementations, we consider the list of squeezing values presented in Figure 3b, which corresponds to a series of faithful values that can be obtained via the Schmidt decomposition of parametric process Hamiltonian of the experiment described in [20,37]. In this work, the input squeezing values for the implementation of the cluster are fixed: this is because our interest lies in understanding what is the best we can do when, like in a realistic situation, we are provided with a given set of squeezed input states. We stress that this scheme, as well as the associated results, is different to the one dealing with the canonical decomposition of [22].

The obtained clusters are optimized, acting on the parameters of the arbitrary orthogonal matrix *O* of Equation (3), by minimizing the fitness function $f\left(\Delta^{2}\delta_{i}\right)=\sum_{i}\Delta^{2}{\bar{\delta }}_{i}$, where $\Delta^{2}{\bar{\delta }}_{i}$ denotes the *i*-th nullifier normalized with the vacuum noise.

As BA, ER, and WS are statistical models for graph families, we implement N=100 graphs for each model with a given set of parameters. We define the average quality of a single graph *j* as $\mu_{j}$ and the average quality for a set of graphs as μ, as follows:

(4) $$\mu_{j}=\frac{1}{48}\sum_{i}\Delta^{2}{\bar{\delta }}_{i,j}\;;\;\mu =\langle \mu_{j}\rangle ,$$

where $\Delta^{2}{\bar{\delta }}_{i,j}$ identifies variance of the *i*-th nullifier of the *j*-th graph. We will indicate as σ the standard deviation of the $\mu_{j}$ values. This way, we can average out the fluctuations due to the randomness of the complex shape.

As we can see from Table 1, the implementation of quantum complex networks following the Barabási–Albert model or the Erdős–Rényi model shows that the quality of the cluster increases when the average number of edges per node, the average degree 〈k〉, increases. The average degree 〈k〉 can be raised by increasing the parameters $m_{BA}$ and $p_{ER}$, for the BA and the ER models, respectively. The results clearly show that the quality of the cluster μ increases with these parameters, until the limiting case of the fully connected graph ($m_{BA}=47$ for the Barabási–Albert model and $p_{ER}=1$ for the Erdős–Rényi model) is reached.

The Watts–Strogatz cluster confirms that the quality of the cluster increases for a larger value of 〈k〉, as we can see by comparing Table 2a,b, but it also shows a peculiar behavior that depends on $p_{WS}$. As already said, $p_{WS}$ is a rewiring parameter that can be varied from 0 to 1 in order to tune the randomness of the graph without changing the average degree, as the number of edges is not changed by the rewiring. From the Table 2, it is clear that the quality of the cluster is reduced when $p_{WS}$ approaches 1, so that regular graphs states are optimized better than random graphs.

**Figure 4:**
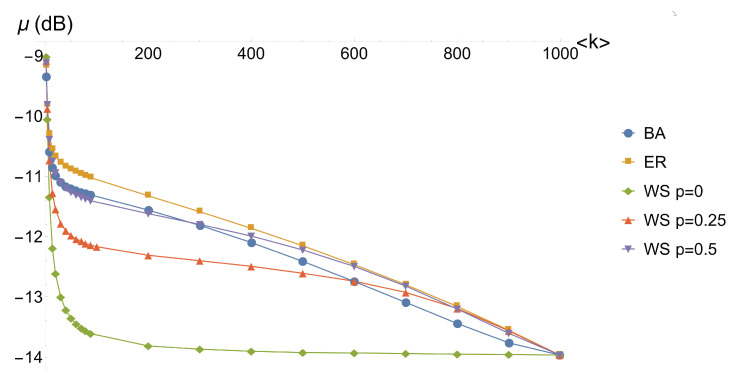
Plot of the mean squeezing value of the nullifiers of the cluster as a function of its average degree 〈k〉 for the different topologies of complex graphs. In the legend, “BA” = Barabási–Albert, “ER” = Erdős–Rényi, “WS p = 0” = Watts–Strogatz with $p_{WS}=0$, “WS p = 0.25” = Watts–Strogatz with $p_{WS}=0.25$ and “WS p = 0.5” = Watts–Strogatz with $p_{WS}=0.5$.

The results we obtained on the 48-node graphs we analyzed show a dependence between the quality of the optimization and the topology of the graph and in particular its dependence on the average degree 〈k〉. In order to reduce the influence of the finite size in the network models we have been using, we repeat the procedure for larger networks. We optimize the mean value μ of nullifiers squeezing (Equation (4)) of a set of 10 complex graphs of 1000 nodes for a certain complex network model with given parameters. The optimization is reiterated by varying the average degree 〈k〉 and by varying the model. The initial list of 1000 squeezing values is obtained from a pseudorandom number generator that created uniformly distributed random numbers in the range [−14,−3]. The results are reported in Figure 4.

The data show that the regular cluster (Watts–Strogatz model with $p_{WS}=0$) converges very fast to its optimal overall quality. Indeed, as we expected from the 48-node cluster analysis, we see that, for a fixed 〈k〉, increasing the regularity, via the $p_{WS}$ parameter, results in a better quality of the optimized cluster and in a faster convergence, as we can see comparing the curves for the Watts–Strogatz model with $p_{WS}=0$, $p_{WS}=0.25$ and $p_{WS}=0.5$. On the other hand, the difference among the Barabási–Albert, Erdős–Rényi and Watts–Strogatz $p_{WS}=0.5$ complex graphs is less significant. Nevertheless, the convergence of the Watts–Strogatz follows a different behavior, being closer to the Barabási–Albert for lower 〈k〉 and converging to the behavior of the Erdős–Rényi for larger 〈k〉. The Erdős–Rényi is found to be the one with the worst overall quality, differing only slightly from the Barabási–Albert behavior.

### 2.3. Concentrating the Squeezing

The overall quality μ is the quantity to optimize if we want to use the cluster in MBQC protocols. On the contrary, if we want to use just two nodes to perform a quantum teleportation protocol, the best we can do is to concentrate the entanglement correlations in the two nodes: this corresponds to concentrating the squeezing on the nullifiers of the two chosen nodes.

Given a set of squeezing values for the input state we use the protocol to concentrate the squeezing on the nullifiers of two nodes $n_{1}$ and $n_{2}$, using the fitness function $f\left(\Delta^{2}{\hat{\delta }}_{i},n_{1},n_{2}\right)=\sum_{i}A_{i}\left(n_{1},n_{2}\right)\Delta^{2}\bar{\delta_{i}}$, where $n_{1}$ and $n_{2}$ are two given modes. We set $A_{i}\left(n_{1},n_{2}\right)=10^{5}$ if $i=n_{1},n_{2}$ and $A_{i}\left(n_{1},n_{2}\right)=1$ otherwise. We see that the squeezing of the nullifiers is indeed concentrated on the two desired nodes, by reaching (and never exceeding) the highest squeezing values provided on the set of input states.

In this case, we will indicate as $\mu_{j}$ the mean of the nodes of the graph *j* that are not concerned with the teleportation protocol and with μ the mean of the $\mu_{j}$ values as follows:

(5) $$\mu_{j}=\frac{1}{46}\sum_{i\neq n_{1},n_{2}}\Delta^{2}{\bar{\delta }}_{i,j}\;;\;\mu =\langle \mu_{j}\rangle ,$$

where $\Delta^{2}{\bar{\delta }}_{i,j}$ identifies variance of the *i*-th nullifier of the *j*-th graph. $\mu_{n_{1}/n_{2}}$ denotes the mean of the set of values $\Delta^{2}{\bar{\delta }}_{n_{1}/n_{2},j}$, where $n_{1}$ and $n_{2}$ are two given nodes on which we chose to perform the teleportation.

As an example, in Table 3, we show that, for a 48-node Barabási–Albert model, the squeezing on the two selected nodes $n_{1}$ and $n_{2}$ indeed take the highest value provided on the input list of Figure 3b. The same results hold for the Erdős–Rényi model and the Watts–Strogatz model.

### 2.4. Creating a Quantum Channel between Nodes by Manipulating Existing Networks

In the previous section, we have seen how to optimize the generation of complex clusters when we have multimode squeezed vacuum modes and we perform linear optics transformations. As already said, several experimental setups demonstrated the ability to deterministically generate large clusters following this approach; therefore, they can be used to generate the complex clusters presented above. The generated cluster can then be distributed among different parties, i.e., different optical modes corresponding to different nodes of the network can be sent to different players, which can eventually use the entanglement correlations between the shared nodes for quantum communication protocols. According to the given task, it may be necessary to reshape the entanglement correlations among the set of nodes. We consider here the simplest case: the protocol wants to establish a maximally entangled state between two arbitrary nodes of a network in order to use it for quantum teleportation. The two-nodes entangled state is a two mode-squeezed state also called EPR, as it is the approximation of the entangled state used by Einstein, Poldolsky, and Rosen in their famous paper in 1935 [38].

The task of generating an entanglement link between chosen nodes corresponds to what is called quantum routing. As already said, the procedure we follow is well suited to CV entangled networks as it is relatively easy to deterministically generate the resources and then reshape them, while in the DV case the generation of optical entangled networks is costly and not deterministic so that the best procedure consists in routing the right entanglement connections at the beginning.

In the following, we reshape the resource by allowing only for the easiest operations that preserve the number of nodes, i.e., passive linear optics transformations. Quadrature measurements for cluster state reshaping are also an option, but this would imply the removal of the measured node and subsequently the owner of said mode could be cut off from the communication network. For this reason, we will concentrate on linear optics operations, and we will leave open the possibility, in future works, of adding measurements if no other option is possible. These can be global, when they operate on all the nodes at the same time, or local, when they act on a subset of nodes.

The case of a global transformation is somewhat trivial as, if we are provided with a cluster *A* implemented with a linear optics transformation $S_{A}$ acting on a set of squeezed input states, it is always possible to find the transformation that leads us to the cluster *B*. This transformation is simply $S=S_{B}\cdot S_{A}^{-1}$, where $S_{B}$ is the linear optics transformation that we should perform on the same set of input states to build the cluster *B*.

**Figure 5:**
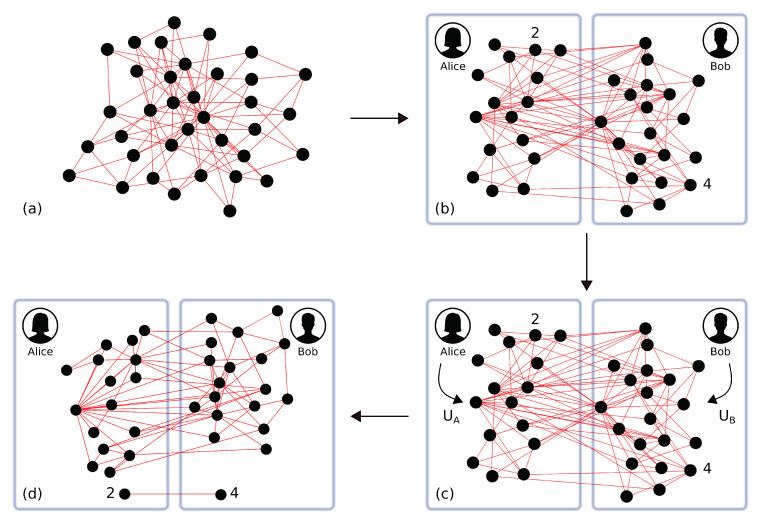
(**a**) a quantum network is created; (**b**) the resource is distributed to two spatially separated teams, Alice and Bob; (**c**) Alice performs a linear optics operation $U_{A}$ on her set of nodes and Bob performs a linear optics operation $U_{B}$ on his set of nodes to create a quantum channel out of two given nodes; (**d**) the quantum channel is established.

We now consider a more interesting scenario: the modes of the cluster are distributed to two spatially separated parties, such that each party is allowed to perform local linear optics transformations only on its set of nodes, as shown in Figure 5. We then want to check which cluster shapes can be reconfigured in order to get a teleportation channel between two arbitrary nodes. A solution to this problem has already been found if we allow for more general symplectic transformations and weighted graphs [39].

Let us say that *n* and *p* are the number of nodes of party A (Alice) and B (Bob), respectively. We want to act with a linear optics transformation $U_{A}$ locally on the *n* modes and with a linear optics transformation $U_{B}$ locally on the *p* modes. The transformation acting on the whole set of quadratures of the modes then reads

(6) S=Re(UA)0−Im(UA)00Re(UB)0−Im(UB)Im(UA)0Re(UA)00Im(UB)0Re(UB),

where $U_{A}$ and $U_{B}$ are two unitary matrices parametrized respectively by $n^{2}$ and $p^{2}$ parameters [40]. A method for the generation of numerically random unitary matrices is presented in [41]. If we define $\sigma_{1}$ as the covariance matrix of the cluster we are given and $\sigma_{2}$ as the covariance matrix of the cluster we obtain after the transformation,

(7) $$\sigma_{2}=S\sigma_{1}S^{T}$$

holds, where *S* is defined in Equation (6). Our goal is to find the two matrices $U_{A}$ and $U_{B}$, whose real and imaginary parts define *S*, such that $\sigma_{2}$ is of the desired form. In our case, we want $\sigma_{2}$ to be such that, for two given nodes, we get an EPR channel. In this case, it is hard to find an analytical solution to the problem, so we use a Derandomized Evolution Strategy (DES) algorithm to explore the parameter space [42].

If $n_{1}$ and $n_{2}$ are the nodes out of which we want to obtain an EPR channel, we can define

(8) $$\begin{array}{c} \sigma_{2,red}\left(n_{1},n_{2}\right)=\left(\begin{array}{c} \sigma_{2,n_{1}}\\ \sigma_{2,n_{2}}\\ \sigma_{2,n_{2}+n+p}\\ \sigma_{2,n_{2}+n+p} \end{array}\right) \end{array}$$

as the reduced 4×2(n+p) matrix obtained by selecting only the rows of $\sigma_{2}$ we want to set as a quantum channel. We thus define the 4×2(n+p) matrix $\sigma_{channel}$ as the matrix with null entries except for

(9) $$\begin{array}{c} \sigma_{1,n_{1}}=\sigma_{2,n_{2}}=\sigma_{3,n_{1}+n+p}=\sigma_{4,n_{2}+n+p}=\alpha , \end{array}$$

(10) $$\begin{array}{c} \sigma_{1,n_{2}+p+n}=\sigma_{2,n_{1}+p+n}=\sigma_{3,n_{2}}=\sigma_{4,n_{1}}=\beta , \end{array}$$

where the subscript “channel” has been omitted for simplicity and where α and β are numerical values that we fix according to the squeezing we want to attain on the modes of the quantum channel. This squeezing cannot exceed the squeezing of the input states of the cluster. For simplicity, we worked with an equal value of squeezing of –10 dB, both for the input squeezed states used to implement the cluster and for the squeezing chosen for the quantum channel. We will search for the minimal value of the function

(11) $$f_{opt}=\|\sigma_{channel}-\sigma_{2,red}\left(n_{1},n_{2}\right)\|,$$

where ‖·‖ indicates the Frobenius norm.

**Figure 6:**
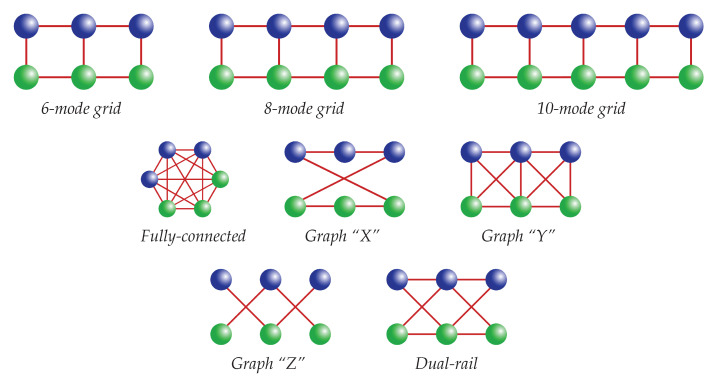
Graphs analyzed with the aim of creating an EPR channel between Alice (green) and Bob (blue) (or eventually between nodes of the same team).

In Table 4, we show preliminary results on different graphs with a restricted number of nodes, shown in Figure 6. For the 6-mode and 10-mode “grid” graphs and the graphs “X” and “Y”, a result can be found for the creation of a quantum channel between Alice and Bob. For these structures, however, it was not possible to find a solution for the creation of a quantum channel between nodes of the same team. The opposite stands for the fully-connected cluster, for which it was possible to create a channel between nodes of the same team but not between nodes of different teams. Lastly, for the graph “Z”, which represents two 3-mode cluster states distributed to the two parties, for the dual-rail and for the 8-mode “grid” graph, no solution was found. As an example, the results of the fully-connected graph and of the 6-mode “grid” graph of Figure 6 are shown in Appendix A.

## 3. Discussion

We have shown that, for CV cluster states with complex graphical representation the quality, measured as the mean of the variance of the nullifiers, can be better optimized when the quantity of entanglement links increases. This is probably due to the fact that, if the number of links is higher, there are more available ways, given by the newly introduced links, of distributing the larger values of the initial squeezing. We have analyzed in the quantum regime different complex shapes corresponding to different models of real-world networks. In the Barabási–Albert and Erdős–Rényi models, regardless of the topology, clusters with a similar average degree 〈k〉 have a comparable overall quality. The average degree 〈k〉 in complex graphs could thus be used as a benchmark for the quality of the state implemented with the optimization protocol. Moreover, analyzing “small-world” networks that evolve from a regular network to a random network as their characteristic parameter $p_{WS}$ increases, we found that randomness in the structure is detrimental to the quality of the state. The optimization procedure can be also used to concentrate the entanglement between two given nodes. Global optimizations of cluster states via linear optics unitaries can always be realized via analytical procedures.

On the contrary, if the cluster is distributed to different locations and the players want to reshape it via local operations (quantum routing) with the aim of performing quantum communication protocols, we need to use a numerical procedure in order to deal with the larger number of constraints. In this case, we have used a Derandomized Evolutionary Strategy (DES) algorithm with a suitable fitness function, the one of Equation (11), in order to find solutions to generate an EPR state between two arbitrary nodes of a network shared between two parties, which can perform only local linear optics operations.

We have studied small networks and we have found that it is possible to create EPR channels between two nodes of the two different teams for some network shapes, while creating an EPR channel from two nodes of the same team has never been possible, except for the case of the fully connected network. Thus, except for this last case, it has never been possible, by using only local linear optics operations, to disconnect two nodes of one player from the ones of the other player. It has to be stressed that, in the cases where no solution is found, we cannot conclude with certainty that the solution does not exist, as the DES algorithm can be stuck in a local minimum in the parameters space.

In future works, we will investigate quantum routing operations when complex clusters are distributed between several parties by also adding, when linear optics operations are not sufficient, quadrature measurements. The measurement of the *q* quadrature of a mode of the cluster allows in fact for node removal, while the measurement of *p* is useful in wire shortening [22]: both can be used in order to cut the residual edges after the optimization via linear optics operations.

## 4. Materials and Methods

To implement the networks and carry out the data analysis, we used Wolfram Mathematica Version 11.3 (Wolfram Research, Champaign, Illinois, USA). In particular, the Barabási–Albert, Erdős–Rényi and Watts–Strogatz models are already embedded in the software. Wolfram Mathematica has been used also to implement the DES *(μ-λ) iso-CMA algorithm* presented in [42]. The goal of a DES algorithm is the optimization of a given function f(x), where x is a vector of parameters. We are free to choose our starting point $x^{old}$ in the parameter landscape, and we “mutate” it, generating λ new points (*offspring*) as

(12) $$x_{k}^{new}=x^{old}+\Delta x_{k},\;\mathrm{where}\;k=1,\cdots ,\lambda ,$$

where $\Delta x_{k}$ is drawn from a multivariate normal distribution $N(0,\sigma^{2}𝟙)$. The λ new points are then evaluated with respect to the chosen fitness function *f* and sorted. The μ “mutants” that provide the best result are chosen to generate a new parent as

(13) $$x^{new}=\sum_{k}w_{k}x_{k}^{new},$$

where $\sum_{k}w_{k}=1$. The procedure is then iterated, setting $x^{new}$ as the new starting point. A learning component is provided by updating, at each generation, the global step-size σ. As already said above, in our case, we want to reach a specific value $f_{opt}∼0$ of a non-negative fitness function. However, it is never guaranteed that the DES procedure finds the global extremum.

## Figures and Tables

**Table 1:** Mean μ and standard deviation σ of the values $\mu_{j}$ of Equation (4) evaluated on N=100 Barabási–Albert (a) and Erdős–Rényi (b) graphs with different characterizing parameters and consequently different average degrees 〈k〉, optimized using the function $f\left(\Delta^{2}\delta_{i}\right)=\sum_{i}\Delta^{2}{\bar{\delta }}_{i}$. Without the optimization protocol, μ takes the value of −3.48 dB for the Erdős–Rényi model, independently of the value of $p_{ER}$, and it oscillates between −3.48 dB and −3.72 dB for the Barabási–Albert model.

(a) Barabási–Albert				(b) Erdős–Rényi			
$m_{BA}$	μ (dB)	[μ±σ] (dB)	〈k〉	$p_{ER}$	μ (dB)	[μ±σ] (dB)	$\bar{\langle k_{j}\rangle }$
1	−4.70	[−4.73,−4.67]	1.96	0.2	−5.50	[−5.54,−5.46]	9.35
5	−5.55	[−5.58, −5.53]	9.38	0.4	−5.80	[−5.83, −5.76]	18.83
10	−5.82	[−5.84, −5.80]	17.71	0.6	−6.02	[−6.04, −6.00]	28.29
20	−6.15	[−6,16, −6.14]	31.25	0.8	−6.22	[−6.23, −6.21]	37.58
47	−6.33	[−6.33, −6.33]	47	1	−6.33	[−6.33, −6.33]	47

**Table 2:** Mean μ and standard deviation σ of the values $\mu_{j}$ of Equation (4) evaluated on N=100 Watts–Strogatz graphs with different parameter $p_{WS}$ and different 〈k〉, optimized using the function $f\left(\Delta^{2}\delta_{i}\right)=\sum_{i}\Delta^{2}{\bar{\delta }}_{i}$. Without the optimization protocol, μ takes the value of −3.48 dB, independently of the value of $p_{WS}$ or 〈k〉.

(a) 〈k〉=4			(b) 〈k〉=8		
$p_{WS}$	μ (dB)	[μ±σ] (dB)	$p_{WS}$	μ (dB)	[μ±σ] (dB)
0	−5.19	[−5.19,−5.19]	0	−5.79	[−5.79,−5.79]
0.1	−5.16	[−5.17,−5.14]	0.1	−5.69	[−5.71,−5.66]
0.4	−5.10	[−5.12,−5.07]	0.4	−5.49	[−5.51,−5.46]
0.7	−5.09	[−5.11,−5.07]	0.7	−5.43	[−5.46,−5.40]
1	−5.09	[−5.12,−5.06]	1	−5.43	[−5.46,−5.41]

**Table 3:** Means $\mu_{12}$, $\mu_{13}$ and μ of the nullifiers of the nodes 12 and 13 and of the value $\mu_{j}$ of Equation (5) evaluated on N=100 Barabási–Albert graphs with different parameter $m_{BA}$, optimized using the function $f\left(\Delta^{2}{\hat{\delta }}_{i}\right)=\sum_{i}A_{i}\Delta^{2}\bar{\delta_{i}}$, where $A_{i}=10^{5}$ if i=12,13 and $A_{i}=1$ otherwise.

$m_{BA}$	$\mu_{12}$ (dB)	$\mu_{13}$ (dB)	μ (dB)
1	−6.51	−6.51	−4.61
5	−6.51	−6.51	−5.48
10	−6.51	−6.51	−5.76
20	−6.51	−6.51	−6.10
47	−6.51	−6.51	−6.32

**Table 4:** Results on the possibility to create a quantum communication channel for the graphs of Figure 6, between nodes belonging to different teams A and B and between nodes belonging to the same team.

Graph	Between A and B	Same Team
6-node grid	Yes	No
8-node grid	No	No
10-node grid	Yes	No
“X”	Yes	No
“Y”	Yes	No
Fully-connected	No	Yes
“Z”	No	No
Dual-rail	No	No

**Figure A1:**
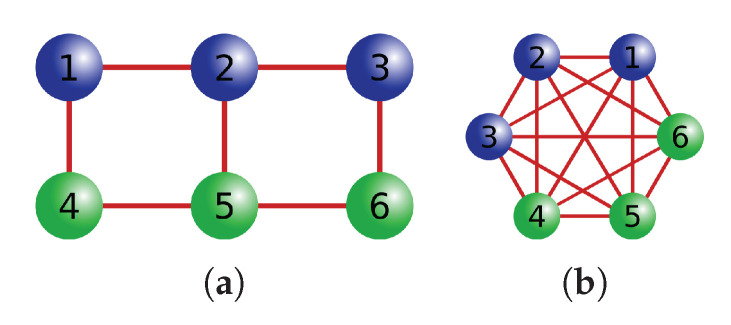
Graphs whose results for the creation of an EPR channel between Alice (green) and Bob (blue) are shown in this Appendix. (**a**) 6-mode grid cluster; (**b**) 6-mode fully connected cluster.

## Appendix A

We will present here the result for the creation of a quantum channel out of two given nodes for the graphs shown in Figure A1.

For the 6-mode “grid” graph of Figure A1a, the suitable unitary matrices $U_{A}$ and $U_{B}$ (see Equation (6)) that we need for the creation of a quantum channel out of the nodes 1 and 4 are

Re(UA)=−0.564055O(10−16)0.5640550.250315O(10−16)0.250315O(10−16)−0.277133O(10−16),Im(UA)=−0.426429O(10−16)0.426429−0.661319O(10−17)−0.661319O(10−16)−0.960831O(10−16)Re(UB)=−0.564055O(10−17)0.564055−0.449914O(10−17)−0.449914O(10−18)−0.993175O(10−17),Im(UB)=0.426429O(10−17)−0.426429−0.545507O(10−18)−0.545507O(10−18)−0.116635O(10−17).

Using these transformations, the correlations between 1 and 4 and the other nodes are at most of the order of $10^{-15}$.

For the “fully connected” graph of Figure A1b, the suitable unitary matrices $U_{A}$ and $U_{B}$ (see Equation (6)) that we need for the creation of a quantum channel out of the nodes 1 and 2 are

Re(UA)=0.56149−0.397134−0.164356−0.561490.3971340.1643560.4082480.4082480.408248,Im(UA)=−0.134394−0.4190680.553462−0.134394−0.4190680.553462−0.408248−0.408248−0.408248Re(UB)=0.4477150.2939870.1043920.4361760.614297−0.4727510.1245020.3612370.860632,Im(UB)=−0.7066390.450256−0.01244740.201155−0.4083780.04074970.232375−0.1903050.152002.

Using these transformations, the correlations between 1 and 2 and the other nodes are at most of the order of $10^{-15}$.
